# Evaluation of deep learning based dose prediction in head and neck cancer patients using two different types of input contours

**DOI:** 10.1002/acm2.14519

**Published:** 2024-09-16

**Authors:** Masahide Saito, Noriyuki Kadoya, Yuto Kimura, Hikaru Nemoto, Ryota Tozuka, Keiichi Jingu, Hiroshi Onishi

**Affiliations:** ^1^ Department of Radiology University of Yamanashi Yamanashi Japan; ^2^ Department of Radiation Oncology Tohoku Univ. Graduate School of Medicine Sendai Japan; ^3^ Radiation Oncology Center Ofuna Chuo Hospital Kamakura Japan

**Keywords:** deep learning based dose prediction, head and neck cancer, volumetric‐modulated arc therapy

## Abstract

**Purpose:**

This study evaluates deep learning (DL) based dose prediction methods in head and neck cancer (HNC) patients using two types of input contours.

**Materials and methods:**

Seventy‐five HNC patients undergoing two‐step volumetric‐modulated arc therapy were included. Dose prediction was performed using the AIVOT prototype (AiRato.Inc, Sendai, Japan), a commercial software with an HD U‐net‐based dose distribution prediction system. Models were developed for the initial plan (46 Gy/23Fr) and boost plan (24 Gy/12Fr), trained with 65 cases and tested with 10 cases. The 8‐channel model used one target (PTV) and seven organs at risk (OARs), while the 10‐channel model added two dummy contours (PTV ring and spinal cord PRV). Predicted and deliverable doses, obtained through dose mimicking on another radiation treatment planning system, were evaluated using dose‐volume indices for PTV and OARs.

**Results:**

For the initial plan, both models achieved approximately 2% prediction accuracy for the target dose and maintained accuracy within 3.2 Gy for OARs. The 10‐channel model outperformed the 8‐channel model for certain dose indices. For the boost plan, both models exhibited prediction accuracies of approximately 2% for the target dose and 1 Gy for OARs. The 10‐channel model showed significantly closer predictions to the ground truth for D50% and Dmean. Deliverable plans based on prediction doses showed little significant difference compared to the ground truth, especially for the boost plan.

**Conclusion:**

DL‐based dose prediction using the AIVOT prototype software in HNC patients yielded promising results. While additional contours may enhance prediction accuracy, their impact on dose mimicking is relatively small.

## 1 INTRODUCTION

Intensity‐modulated radiotherapy (IMRT) is widely used in patients with head and neck cancer (HNC). IMRT is administered via two approaches, which include the lymph node area as a preventive measure, namely the simultaneous integrated boost (SIB)^1^, ^2^ and sequential boost (two‐step) methods.^3^, ^4^ Some reports have compared these treatment plans^5^, ^6^, ^7^ and their outcomes.^8^, ^9^, ^10^ However, the decision making for implementation of a suitable method to treat patients with HNC remains with individual institutions.

All IMRT plans for HNC require inverse planning, where balancing the time spent on treatment planning with the quality of execution is crucial.^11^ In clinical practice, plans must meet specific quality standards like dose constraints at each institution, despite time constraints. Additionally, evaluating the robustness and spatial distribution of the dose on computed tomography (CT) images is also necessary.^12^ Although a treatment planning system (TPS) to quantitatively evaluate the quality of each plan has not yet been implemented, the quality of the plan currently varies among planners and institutions.^13^, ^14^, ^15^, ^16^ Nelms et al. evaluated 125 prostate cancer IMRT treatment plans using a plan quality metric and reported a large variation in the quality of treatment plans regardless of the background factors of the treatment planners.^13^ From another perspective, dummy runs at the time of enrollment in various clinical trials have been achieved to ensure the quality of the plan by identifying variations among sites in advance and providing guidance for deviant cases.^14^, ^15^, ^16^ Therefore, ensuring the quality of treatment planning in IMRT is indeed an important issue in modern radiotherapy.

In recent years, numerous studies have been conducted which introduce knowledge‐based IMRT treatment planning.^17^ In particular, deep learning based methods for predicting IMRT treatment plan dose distributions have been extensively reported.^18^, ^19^, ^20^, ^21^ This method can predict the appropriate dose distribution for contours on CT images by inputting new contours on these images into a prediction model previously trained using a pair of contour information and dose distribution. Nguyen et al. proposed a U‐net‐based 3D voxel‐level dose prediction method for patients with prostate cancer and evaluated its prediction accuracy. The prediction error of the organs at risk (OARs) dose using this approach was < 5%.^22^ Gronberg et al. developed a 3D densely connected U‐Net with dilated convolutions to predict the dose distribution in an SIB plan for HNC.^23^ Their approach achieved prediction accuracy within 3% for the target dose. Hu et al. reported the prediction accuracy of a transformer‐based dose distribution prediction method for SIB plans for HNC.^23^ The dose‐volume histogram (DVH) index showed a prediction error of 2.25% for the target dose. Such dose distribution prediction is expected to improve the efficiency of treatment planning as well as its quality.^24^

There have been many studies on dose prediction, including open‐access contests.^25^ However, the effects of different types of input contours used in model construction on prediction accuracy have not yet been clarified. Particularly, whether information input should include only the target and OAR contour, or whether additional contours are required, remains to be examined. Furthermore, although there have been numerous reports on dose distribution prediction in HNC using SIB methods,^18^, ^25^, ^26^, ^27^, ^28^ studies on the two‐step method are limited.

Therefore, this study aimed to evaluate deep learning based dose prediction methods for patients with HNC using two different types of input contours. These studies were performed using the new commercial dose prediction software AIVOT (prototype version; AiRato. Inc, Sendai, Japan).^29^

## 2 MATERIALS AND METHODS

### 2.1 Ethics approval

This study was approved by the institutional review board of the University of Yamanashi (receipt number: CS0010).

### 2.2 Treatment planning for patients with HNC

This study included 75 patients with HNC who were treated with volumetric‐modulated arc therapy (VMAT) at our hospital. The treatment plan data were used for the initial plan (46 Gy/23 Fr, planning target volume [PTV] D95% prescription) and boost plan (24 Gy/12 Fr, PTV D95% prescription) of the two‐step method. It is noted that this study defines the PTV excluding air within it. In all cases, the initial and boost CT images differed. The dose constraints were PTV (body cut) D95% ≥70 Gy (100%), D2% < 77 Gy (110%), spinal cord Dmax < 50 Gy, brainstem Dmax < 54 Gy, parotid Dmean < 24 Gy (as low as possible), submandibular Dmean < 38 Gy (as low as possible), mandible D2% < 70 Gy, and oral cavity Dmean < 30 Gy (as low as possible). The treatment plan was designed to meet these criteria when the initial and boost doses were combined. The treatment machine was Elekta Synergy with an Agility gantry head (Elekta AB, Stockholm, Sweden), and the TPS was RayStation ver. 10A (RaySearch Laboratories, Stockholm, Sweden). The dose calculation algorithm was a collapsed cone (version 5.3), and the dose calculation grid size was 2 mm. The initial plan was created using a 3‐full arc VMAT, and the boost plan was created using a 1−2 arc VMAT. All plans were created with an arbitrary collimator angle range of 5−355 degrees. Treatment planning was performed by a single experienced medical physicist.

### 2.3 AIVOT prototype

The AIVOT prototype, currently available exclusively for educational and research use as “RatoGuide” (AiRato. Inc, Sendai, Japan), is a software designed to predict dose distribution by inputting planning CT images, RT structures of targets and normal organs, as well as the prescribed doses. For more information on the prediction models used in this study, please refer to the sections “The Prediction Models” and “Model Training and Evaluation.”

After the dose prediction, the predicted doses or dose‐based structures can be exported in DICOM format to third‐party vendor TPS. These dose‐based structures, once imported into TPS, can be utilized to create a deliverable plan using inverse planning with vendor‐supplied objective functions. RayStation was used in this study. For further details, please refer to the section “Dose Mimicking” and Supplementary Document 1.

### 2.4 The prediction models

**FIGURE 1:**
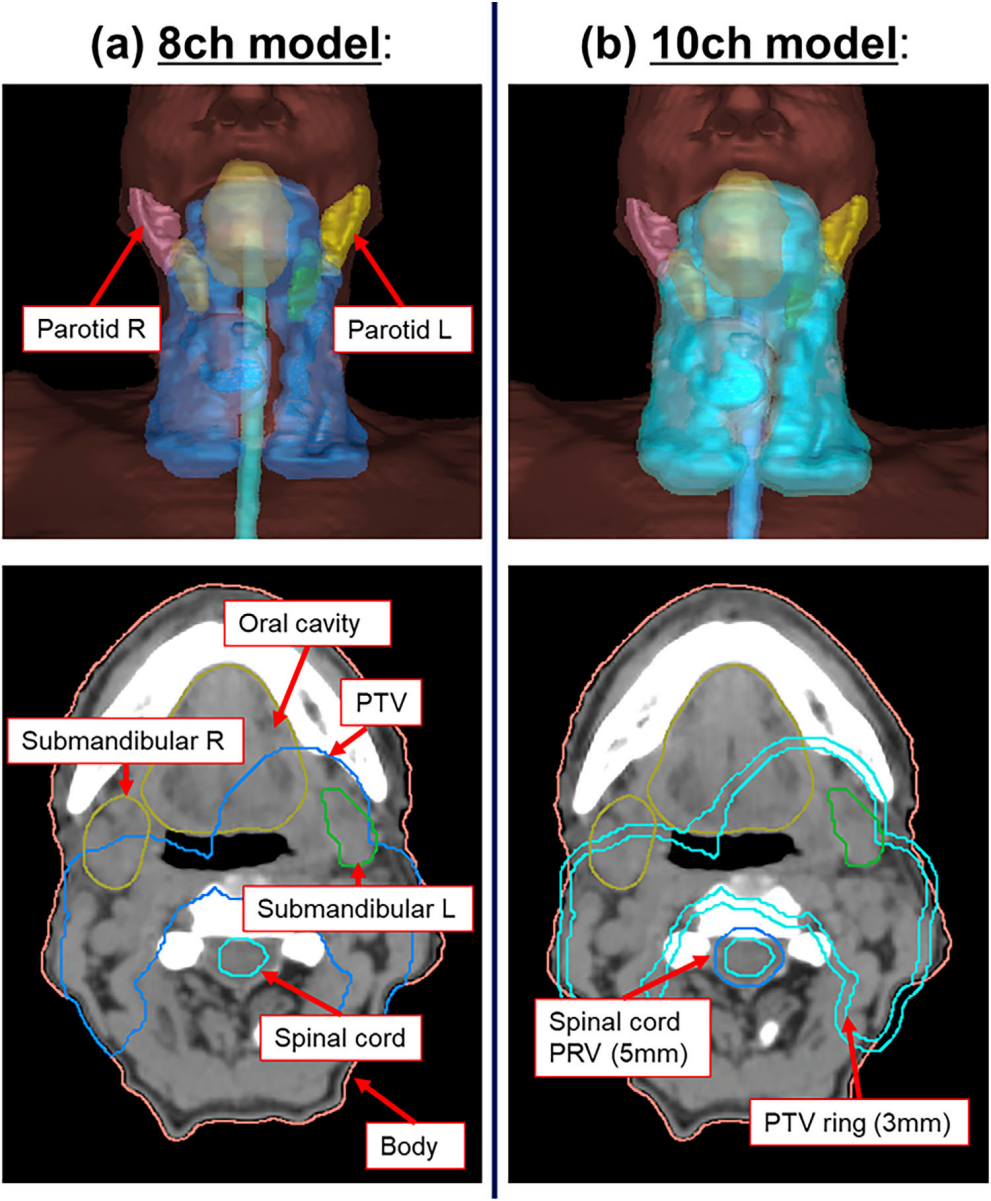
An overview of the two input models [(a) 8‐ch model, and (b) 10‐ch model]. (a) has a total of eight contours: one target [PTV (body cut)] and seven normal tissues (Oral cavity, Parotid L, Parotid R, Submandibular L, Submandibular R, Spinal cord, and Body). (b) has two additional dummy contours: the PTV ring (3 mm ring circumscribing the PTV) and the spinal cord PRV (5 mm expansion from the spinal cord).

Deep learning based dose prediction was performed using the AIVOT prototype, which is capable of HD‐UNet‐based 3D dose prediction, as proposed by Nguyen et al.^30^ Two input models were created as follows: first, with eight contours (8‐channel [8‐ch] model), and the second with 10 contours (10‐channel [10‐ch] model) as the input. Figure 1 presents an overview of the two input models evaluated in this study. In the 8‐ch model, a total of eight contours were used as input information: one target (PTV) and seven normal tissues (oral cavity, left parotid gland [parotid L], right parotid gland [parotid R], left submandibular gland [submandibular L], right submandibular gland [submandibular R], spinal cord, and body). Subsequently, in the 10‐ch model, in addition to the contours of the 8‐ch model described above, two additional dummy contours were used: the PTV ring (3 mm ring circumscribing the PTV) and the spinal cord planning risk volume (PRV, 5 mm expansion from the spinal cord). The inclusion of the PTV ring was aimed at potentially improving the prediction accuracy of the target conformity index (CI), which is critical for effective treatment planning. The expanded spinal cord PRV was selected with the goal of enhancing the prediction accuracy for the maximum dose received by the spinal cord, a significant organ at risk.

### 2.5 Model training and evaluation

**FIGURE 2:**
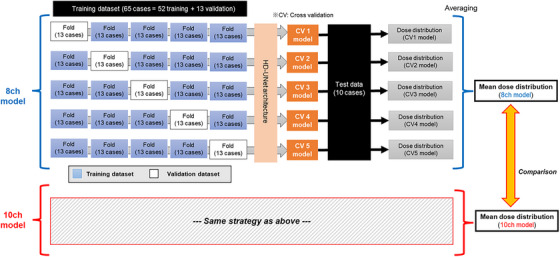
Evaluation workflow in this study. Five CV models were created using 65 cases (52 training + 13 validation). Then, the test data were predicted using these 5 CV models, and the mean dose distribution was generated to compare the 8‐ch model with the 10‐ch model.

The methodological procedure used in this study is illustrated in Figure 2. Of the 75 cases, 65 were used in the model training process and 10 were set aside for the final testing process. It was noted that the number of cases in this study was determined by the available cases at our institution. The number of cases in this study is equal to or greater than the number of cases in previous literature.^31^ Furthermore, the number used for training is defined for adaptation to five‐fold cross validation (CV), with each model utilizing 13 cases. Contour information for the PTV and OARs, along with the dose distributions for 65 cases, were provided to the vendor, and model training was performed. During the training process, a five‐fold CV was performed by dividing the 65 cases into 52 cases for training and 13 cases for validation. Finally, we received the trained models from the vendor and analyzed the test dataset using the AIVOT software.

The software was equipped with five trained models obtained by five‐fold CV, each of which predicted a 3D dose distribution from the input binary masks of the PTV and OARs. The five generated dose distributions were averaged and normalized to D95% = 100% for the PTV. Based on the final prediction result, the accuracy of the dose distribution predictions was evaluated by analyzing the DVH metric, as described below.

These evaluations were executed for both the initial and boost plans, and the results from the 8‐ and 10‐ch models were compared.

### 2.6 Dose mimicking

In the present study, dose mimicking in RayStation was carried out to have the predicted dose distribution used to create a realistic deliverable plan: for the initial plan and the boost plan, 8 and 10‐ ch dose mimicking was carried out, respectively. The beam geometry was the same as for the manual treatment plan. For optimization conditions, please refer to Supplementary document 1.

### 2.7 Dosimetric comparison

The accuracy of the dose distribution prediction was evaluated by comparing the dose volume indices (DVIs). The DVIs for each contour (PTV [D98%, D95%, D50%, D2%, and Dmean], oral cavity [Dmean], parotid L [Dmean], parotid R [Dmean], spinal cord [Dmax], submandibular L [Dmean], and submandibular R [Dmean]) were evaluated by comparing the ground truth (GT) and predicted doses. It is noted that the GT is defined as the manual planning dose distribution.

To evaluate the test set, in addition to the DVIs above, the CI was also calculated using the following formula 1:

(1) $$\mathrm{CI}\;=\frac{TV_{RI}}{TV}\;\times \frac{TV_{RI}}{V_{RI}}$$

Where, $TV_{RI}$ was the target volume covered by the reference isodose, TV was the target volume, and $V_{RI}$ was the volume of the reference isodose. The reference isodoses were 46 Gy in the initial plan and 24 Gy in the boost plan. The mean absolute error (MAE) was calculated for DVIs as follows:

(2) $$\begin{array}{c} \mathrm{MAE}\;=\frac{1}{n}\;\sum_{i}^{n}\left|\left(DVI_{ground\;truth}^{i}-DVI_{prediction}^{i}\right)/D_{prescription}\right| \end{array}$$

Where, *n* was the number of test data points, $D_{\mathrm{prescription}}$ was the prescription dose (46  or 24 Gy), $DVI_{\mathrm{ground}\;\mathrm{truth}}$ was the DVI of the clinical plan, and $DVI_{\mathrm{prediction}}$ was the predicted DVI of the predicted doses (8 or 10‐chl model).

All statistical analyses were performed using the JMP ver.17 (SAS Institute, Cary, NC, USA) software with the Wilcoxon test at a significance level of 0.05.

## 3 RESULTS

Table 1 lists the prediction results of the initial plan for each contour of the 8 and 10‐ch models. No significant differences were found between 8 and 10‐ch for most DVIs of the PTV. However, significant differences were found for D2% and CI of PTV between the GT and each prediction model. For normal organs, there were no significant differences between each prediction model and the GT for most DVIs. On the other hand, significant differences were found only for bilateral submandibular glands. For DVIs of the oral cavity and submandibular R, the 10‐ch model was closer to the GT than the 8‐ch model. For the deliverable plan, significant reductions in CI compared to GT were observed for each of the 8 and 10‐chs, but no significant differences were observed for the other DVIs of the PTV. For normal organs, significant differences from GT were observed in almost DVIs. No significant differences between 8 and 10‐ch models were observed.

**TABLE 1:** Dose volume indices (DVIs) and each *p*‐value for initial plans.

			Predicted dose		*p‐value* (predicted dose)			Deliverable dose		*p‐value* (deliverable dose)		
		Ground truth	8Ch model	10Ch model	GT vs. 8ch	GT vs. 10ch	8ch vs. 10ch	8Ch model	10Ch model	GT vs. 8ch	GT vs. 10ch	8ch vs. 10ch
PTV	*D_98%_*	44.31 ± 0.4	44.5 ± 0.18	44.51 ± 0.2	0.32	0.28	0.77	44.55 ± 0.35	44.57 ± 0.36	0.11	0.11	0.63
	*D_95%_*	46 ± 0	46 ± 0	46 ± 0	―	―	―	46 ± 0	46 ± 0	―	―	―
	*D_50%_*	47.78 ± 0.24	47.66 ± 0.18	47.66 ± 0.19	0.06	0.13	0.85	47.58 ± 0.15	47.57 ± 0.2	0.85	0.06	0.63
	*D_2%_*	48.74 ± 0.29	48.16 ± 0.18	48.13 ± 0.18	<0.01^*^	<0.01^*^	0.43	49.01 ± 0.18	48.96 ± 0.27	0.06	0.19	0.28
	*D_mean_*	47.55 ± 0.24	47.4 ± 0.16	47.39 ± 0.17	0.02^*^	0.03^*^	0.92	47.46 ± 0.13	47.44 ± 0.17	0.03^*^	0.28	0.56
	*CI*	0.83 ± 0.01	0.86 ± 0.01	0.86 ± 0.01	<0.01^*^	<0.01^*^	0.16	0.78 ± 0.02	0.78 ± 0.03	<0.01^*^	<0.01^*^	0.70
Oral cavity	*D_mean_*	27.97 ± 10.07	26.99 ± 10.53	27.47 ± 10.59	0.16	0.32	<0.01^*^	28.21 ± 10.16	28.67 ± 10.17	0.85	0.85	0.08
Parotid L	*D_mean_*	18.86 ± 2.15	18.45 ± 2.47	18.52 ± 2.69	0.28	0.43	0.32	20.08 ± 2.63	20.13 ± 2.82	<0.01^*^	<0.01^*^	0.70
Parotid R	*D_mean_*	17.63 ± 2.24	17.98 ± 1.8	17.77 ± 1.83	0.77	0.92	0.06	19.9 ± 1.62	19.69 ± 1.66	<0.01^*^	<0.01^*^	0.23
Spinal cord	*D_max_*	27.36 ± 4.67	25.9 ± 2.27	26.32 ± 3.26	0.23	0.19	0.63	33.5 ± 2.03	33.61 ± 2.34	<0.01^*^	<0.01^*^	0.38
Submandibular L	*D_mean_*	37.43 ± 7.07	39.71 ± 6.74	39.91 ± 6.55	0.03^*^	0.03^*^	0.08	41.76 ± 5.55	41.91 ± 5.65	0.02^*^	0.02^*^	0.38
Submandibular R	*D_mean_*	34.77 ± 7.25	37.91 ± 6.07	37.71 ± 6.13	0.03^*^	0.06	0.05^*^	40.63 ± 4.76	40.21 ± 5.11	<0.01^*^	<0.01^*^	0.08

Abbreviations: 8‐ch, 8‐ch prediction model; 10‐ch, 10‐ch prediction model; CI, conformity index; *D*_max_, maxmum dose; *D*_mean_, mean dose; *D*_XX%_ = dose to XX% at the target volume; GT, ground truth; PTV, planning target volume.

* *p‐value* < 0.05.

Table 2 presents the results of the boost plan predictions. The mean results of the five‐fold CV for each contour of the 8 and 10‐ch models, as well as the results of the ensemble model are shown. Although there were no significant differences between the 8 and 10‐ch models for most of the DVIs of the PTV, significant discrepancies were identified between the GT and the 8‐ch model (D2%) and between the 8 and 10‐ch models (CI). In addition, D50% and Dmean were significantly close to GT in the 10‐ch model. For normal organs, there were no significant differences between each predictive model and the GT for all the DVIs. For the DVI of the parotid R, the 10‐ch model was significantly closer to the GT. The deliverable plan revealed few significant differences in the DVI of both PTVs and normal organs compared to GTs. Additionally, no significant differences were observed between 8 and 10‐ch models.

**TABLE 2:** Dose volume indices (DVIs) and each *p*‐value for boost plans.

			Predicted dose		*p‐value* (predicted dose)			Deliverable dose		*p‐value* (deliverable dose)		
		Ground truth	8Ch model	10Ch model	GT vs. 8ch	GT vs. 10ch	8ch vs. 10ch	8Ch model	10Ch model	GT vs. 8ch	GT vs. 10ch	8ch vs. 10ch
PTV	*D_98%_*	23.33 ± 0.59	23.62 ± 0.18	23.58 ± 0.2	0.08	1.00	0.08	23.4 ± 0.39	23.36 ± 0.41	0.92	1.00	0.04^*^
	*D_95%_*	24 ± 0	23 ± 0	23 ± 0	―	―	―	24 ± 0	24 ± 0	―	―	―
	*D_50%_*	24.8 ± 0.5	24.67 ± 0.18	24.7 ± 0.18	0.70	0.92	<0.01^*^	24.71 ± 0.22	24.64 ± 0.2	1.00	0.32	0.06
	*D_2%_*	25.33 ± 0.57	24.94 ± 0.26	24.97 ± 0.23	0.05^*^	0.06	0.16	25.42 ± 0.36	25.27 ± 0.35	0.23	1.00	0.04^*^
	*D_mean_*	24.71 ± 0.42	24.94 ± 0.26	24.61 ± 0.15	0.49	0.92	<0.01^*^	24.66 ± 0.19	24.59 ± 0.18	0.85	0.38	0.06
	*CI*	0.82 ± 0.08	0.89 ± 0.02	0.89 ± 0.02	<0.01^*^	<0.01^*^	0.38	0.84 ± 0.04	0.84 ± 0.04	1.00	0.63	0.23
Oral cavity	*D_mean_*	10.21 ± 7.81	10.18 ± 7.9	10.13 ± 7.88	0.85	0.77	0.13	10.05 ± 8.04	9.96 ± 7.95	0.56	0.56	0.23
Parotid L	*D_mean_*	5.62 ± 4.35	5.25 ± 4.6	5.14 ± 4.49	0.13	0.11	0.07	5.12 ± 4.52	4.93 ± 4.38	0.06	0.02^*^	<0.01^*^
Parotid R	*D_mean_*	4.39 ± 3.28	3.62 ± 2.55	3.89 ± 2.72	0.06	0.43	0.02^*^	3.71 ± 2.59	3.9 ± 2.73	0.16	0.32	0.05^*^
Spinal cord	*D_max_*	10.78 ± 2.83	9.89 ± 0.86	9.72 ± 0.85	0.38	0.19	0.19	10.82 ± 1.43	10.58 ± 1.35	0.38	0.56	0.19
Submandibular L	*D_mean_*	13.98 ± 7.62	14.08 ± 7.5	13.99 ± 7.49	1.00	0.70	1.00	13.69 ± 7.58	13.6 ± 7.58	0.28	0.13	0.38
Submandibular R	*D_mean_*	13.06 ± 6.44	13.39 ± 7.37	13.64 ± 7.36	1.00	0.85	0.08	13.15 ± 7.49	13.34 ± 7.34	0.92	1.00	0.38

Abbreviations: 8‐ch, 8‐ch prediction model; 10‐ch, 10‐ch prediction model; CI, conformity index; *D*_max_, maxmum dose; *D*_mean_, mean dose; *D*_XX%_ = dose to XX% at the target volume; GT, ground truth; PTV, planning target volume.

* *p‐value* < 0.05.

**FIGURE 3:**
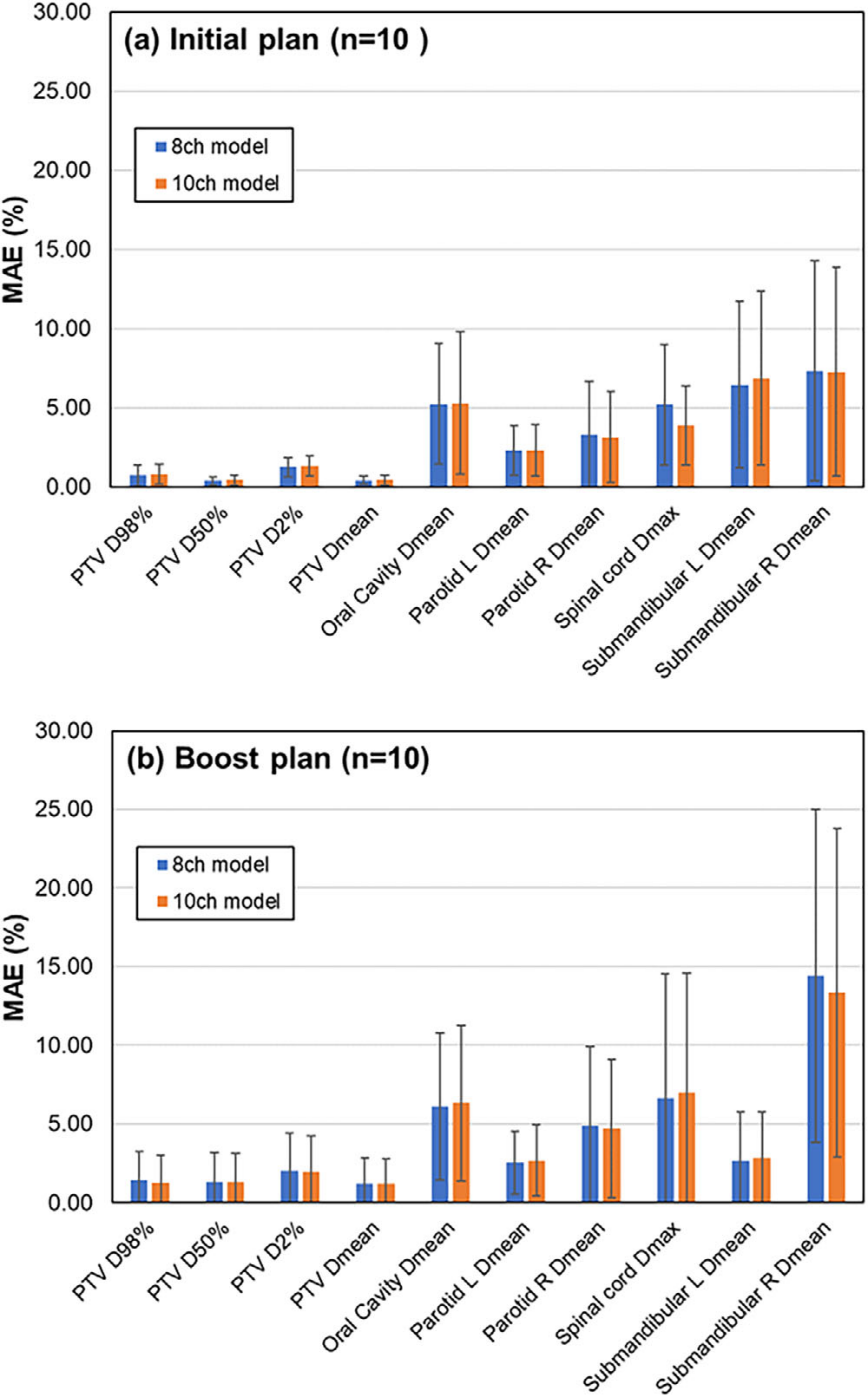
Mean absolute error (MAE) for each dose volume index (DVI) (Ensemble model, average of all 10 cases). (a) and (b) show the results of the initial plan and boost plan, respectively, for the 8‐ch model (blue) and the 10‐ch model (orange). The error bar indicates 1 standard deviation (SD).

Figure 3 shows the MAE results. For the initial plan, the DVIs of the PTV were within 1.33% for both the 8 and the 10‐ch models. In addition, the OARs were within 5.30%, 3.32%, 5.32%, and 7.33% for the oral cavity, bilateral parotid, spinal cord, and bilateral submandibular glands, respectively. For the boost plan, the DVIs of the PTV were within 2.01% for both the 8 and 10‐ch models. Meanwhile, the OARs were within 6.32%, 4.68%, 6.95%, and 14.42% for the oral cavity, bilateral parotid glands, spinal cord, submandibular, and bilateral submandibular glands, respectively.

**FIGURE 4:**
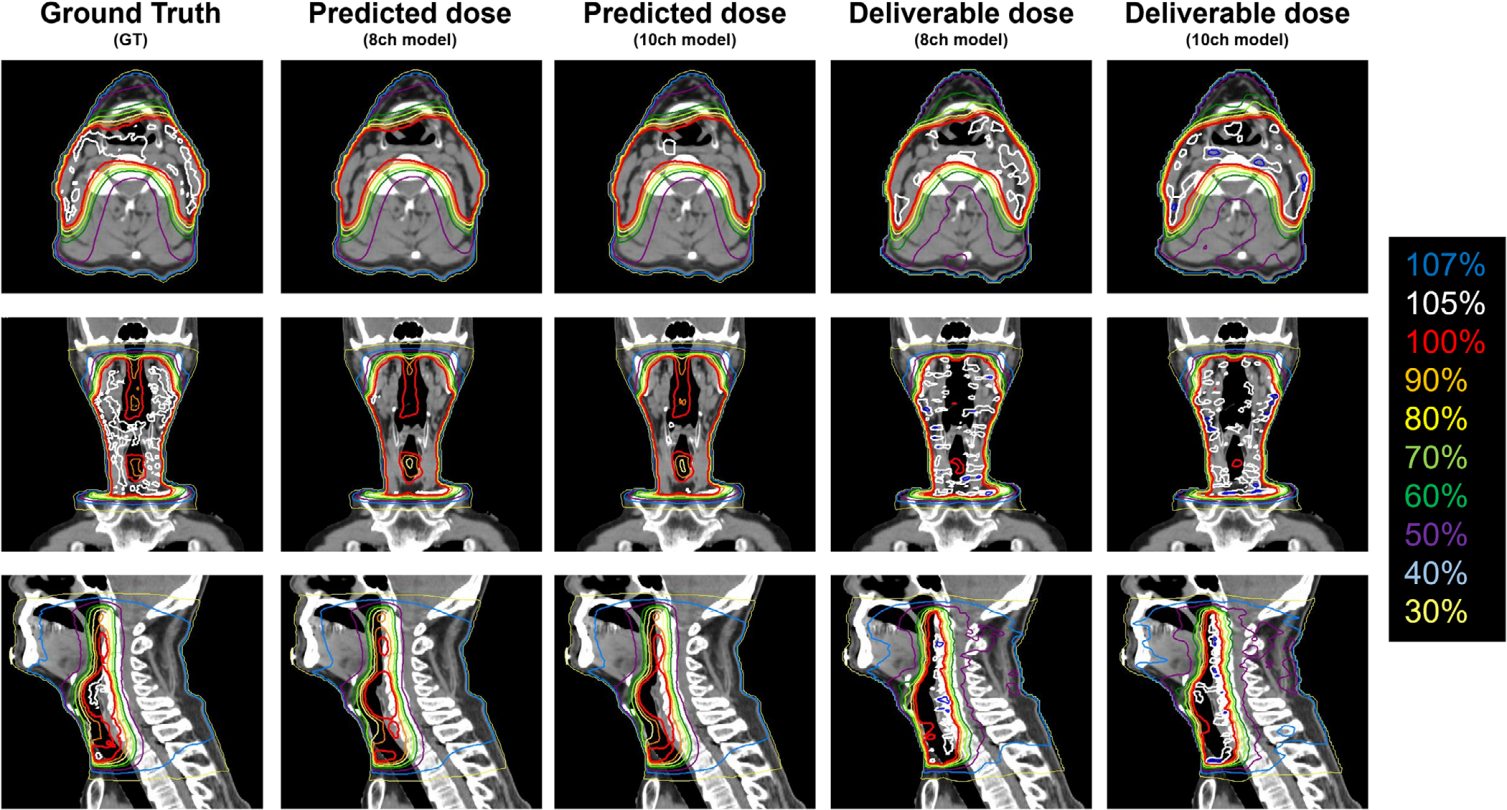
The results of a typical case for initial dose distribution (46 Gy/23 Fr). Displayed in sequence from left to right are Ground Truth (GT), predicted dose with the 8‐ch model, predicted dose with the 10‐ch model, deliverable dose with the 8‐ch model, and deliverable dose with the 10‐ch model. Images of the axial, coronal, and sagittal planes are, respectively, shown.

**FIGURE 5:**
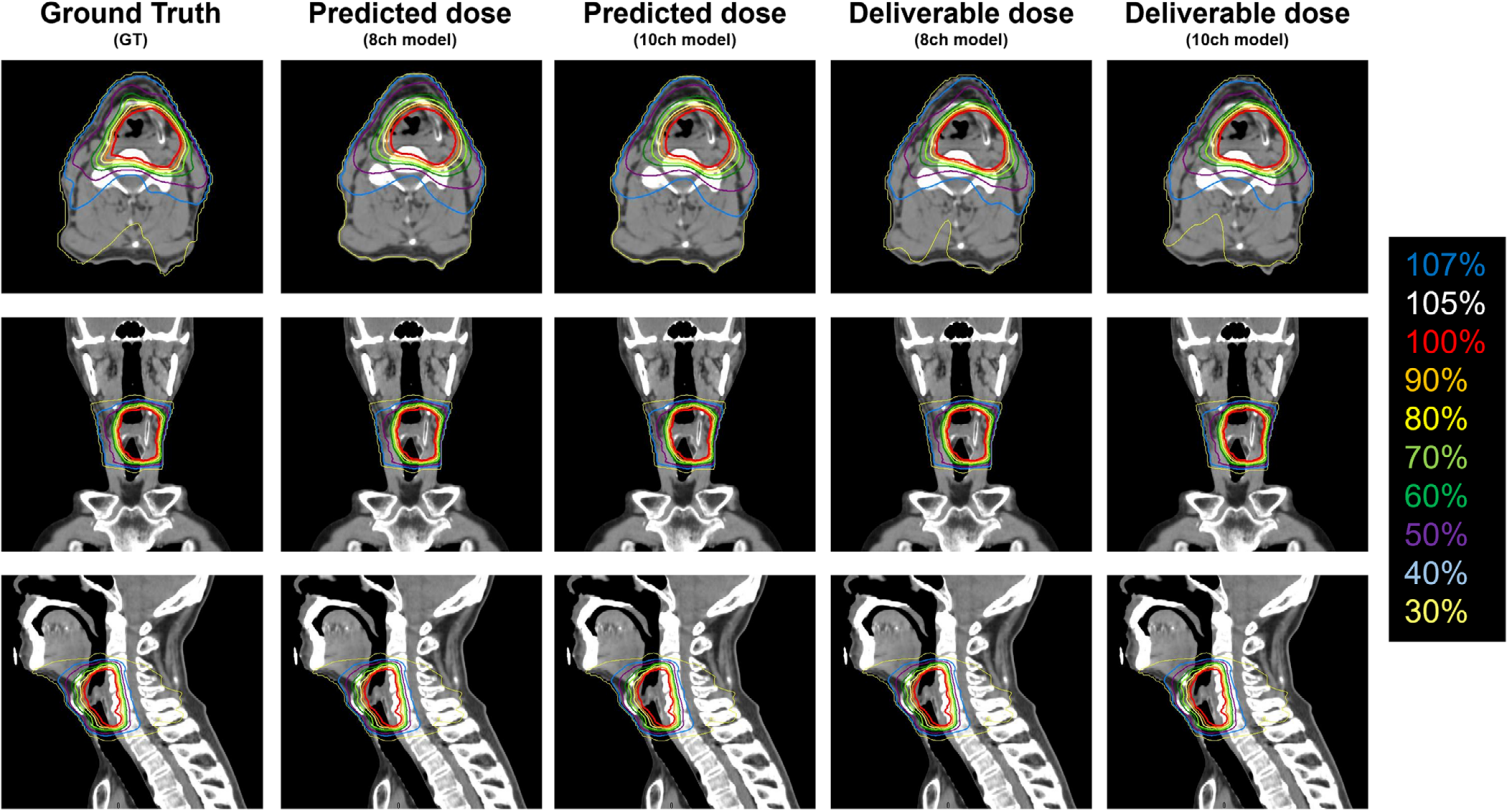
The results of a typical case for boost dose distribution (24 Gy/12 Fr). Displayed in sequence from left to right are Ground Truth (GT), predicted dose with the 8‐ch model, predicted dose with the 10‐ch model, deliverable dose with the 8‐ch model, and deliverable dose with the 10‐ch model. Images of the axial, coronal, and sagittal planes are, respectively, shown.

Similar trends were observed for each predictive and deliverable dose for most cases. A representative example of the initial treatment plan (46 Gy) is shown in Figure 4. The sequence from left to right includes GT, predicted dose with the 8‐ch model, predicted dose with the 10‐ch model, deliverable dose with the 8‐ch model, and deliverable dose with the 10‐ch model. For the predicted dose, the intensity of hot spots within the target area was diminished relative to the GT, while the low and medium dose ranges were achieved with no discernible variation. On the other hand, there was a slight difference in the dose inside and outside the target in the deliverable plan (more hot spots inside the target and a locally larger spread of low and medium doses). A representative example of a Boost plan (24 Gy) is also shown in Figure 5. In this case, the variances among GT, predicted dose, and deliverable dose were remarkably slight.

## 4 DISCUSSION

In recent years, there have been many studies on predicting dose distribution from CT images and contour information,^26^, ^27^, ^28^, ^31^, ^32^, ^33^ along with competitive challenges for models to standardize the technique.^25^ For instance, in the OpenKBP challenge, Gronberg et al. reported that the predicted normalized target DVH metrics were within 3% of the clinical plans, and the predicted OAR DVH metrics were within 2 Gy of the clinical plans.^18^ They used the SIB method, however, our study focused on the two‐step method for treatment of patients with HNC using the AIVOT prototype. To the best of our knowledge, there have been no reports on dose prediction for HNC using commercial systems such as the AIVOT prototype, and we believe that this study will contribute to the development of the new research field on dose prediction in the future. We achieved a prediction accuracy of approximately 2% for the target dose and an accuracy within 2 Gy for the OARs, with some exceptions. Thus, our study demonstrates for the first time that even with the two‐step method, high accuracy in dose prediction can be achieved. In both the 8 and 10‐ch models, the prediction accuracy tended to be poorer for the boost plan than for the initial plan. This may be attributed to the increased distance between the OARs and the target in the boost plan and the varying degrees of dose reduction.

Moreover, there have been no reports evaluating the differences in input contours. In this study, we compared a model with 8 contour inputs to a model with 10 contour inputs. On comparison, these two models had no significant differences for most dose indices. However, for some dose indices, the 10‐ch model was significantly closer to the GT. This may be due to the fact that the added contours mimic the dose distribution more locally. The use of dummy contours is typically effective in IMRT optimization calculations, and our results suggest that the use of dummy contours may be effective in deep learning‐based dose prediction as well.

Our results showed that the prediction errors for the submandibular glands and oral cavity tended to be relatively larger than those for other OARs, such as the parotid glands. This trend deviates from the patterns observed in previous studies.^18^, ^23^ This may have been due to the treatment planning policies of the dataset used in this study. The treatment plans were created such that the dose to each OAR not only met certain criteria, but also such that the mean doses were as low as possible. In the case of the parotid gland, dose reduction is a simple trade‐off with PTV dose coverage. However, because the submandibular gland and oral cavity are located close to each other, reducing the dose to one may result in a higher dose to the other. This complex trade‐off, including PTV dose coverage, may have led to the formation of complex dose distributions from plan to plan and consequently reduced the accuracy of dose distribution predictions for those OARs. Rechecking and refining the quality of treatment plans is an important and interesting next step for creating a more practical dose distribution prediction model.

Dose mimicking was also implemented for the predicted dose distribution in this study. No distinction was observed between the 8 and 10‐ch model configurations. This indicates that although some discrepancies were noted in the predicted dose, these differences were effectively neutralized during the optimization process for the deliverable dose, resulting in no significant variance.

There were four limitations in this study. First, no prospective planning based on the predicted dose distribution was conducted. In other words, whether the predicted dose distribution can be reproduced in clinical practice was not examined, which is a subject for future studies. Second, the differences in the input contours were slight (only two, the PTV ring and spinal cord PRV), and the influence of the other dummy contours was not evaluated. The effectiveness of these contours is inherently dependent on the specific positional relationships between the PTV and adjacent normal organs. Third, since the models in this study were developed using data from a single institution, their applicability across multiple institutions remains uncertain. Different treatment planning policies may require reassessing the model at each facility to determine its clinical usefulness. Fourth, increasing the number of cases used in the prediction model may enhance its accuracy. These issues should be addressed in future studies.

In conclusion, we evaluated the differences in the dose distribution prediction method of the two‐step method in patients with HNC implemented in the AIVOT prototype when two different contours were input. The accuracy of dose prediction by deep learning in the two‐step method for treatment of patients with HNC was confirmed to be comparable to that of previous studies. The 10‐ch prediction model may be more effective than the 8‐ch prediction model in ensuring the target dose and reducing the OAR dose for certain dose indices. Particularly, the addition of a ring‐shaped ROI adjacent to the target may be effective in improving the prediction of the target dose.

## AUTHOR CONTRIBUTIONS

Study conception and design: Masahide Saito. Noriyuki Kadoya, and Yuto Kimura

Analysis and interpretation of data: Masahide Saito, Yuto Kimura, Hikaru Nemoto, and Ryota Tozuka

Drafting of manuscript: Masahide Saito and Yuto Kimura

Critical revision: Masahide Saito, Noriyuki Kadoya, Yuto Kimura, Keiichi Jingu, and Hiroshi Onishi.

## CONFLICT OF INTEREST STATEMENT

Noriyuki Kadoya, Yuto Kimura, and Ryota Tozuka owned stock in AiRato Inc., while Masahide Saito and Hiroshi Onishi received grants from AiRato Inc.

## Supporting information



Supplementary information
