# Supplementary material for: Evaluation of deep learning based dose prediction in head and neck cancer patients using two different types of input contours
Source: J Appl Clin Med Phys. 2024 Sep 16;25(12):e14519. doi: 10.1002/acm2.14519 (PMC11633794; doi:10.1002/acm2.14519)
Supplement: Supplementary file 1 — Supplementary information [file ACM2-25-e14519-s001.docx]

**Supplemental document 1**

This document presents the dose mimicking methods implemented in RayStation. The treatment plans were devised using four rounds of optimization for the initial plan and six rounds for the boost plan. Throughout all rounds of optimization, the objective function and constraints remained consistent. All these procedures were executed entirely automatically using an IronPython script.

**A. Optimization settings for initial plans**

1^st^ round

- Optimization tolerance: 1.000E-9
- Max number of iterations:400
- Iteration before conversion:100
- Compute intermediate dose = ON
- Compute final dose = ON

2^nd^ round

- Optimization tolerance: 1.000E-9
- Max number of iterations:200
- Iteration before conversion:100
- Compute intermediate dose = ON
- Compute final dose = ON

Scaled dose

- ROI = Dose line 100%RX
- Dose at volume = 98%
- Dose = 4600

3^rd^ round

- Optimization tolerance: 1.000E-9
- Max number of iterations:400
- Iteration before conversion:100
- Compute intermediate dose = ON
- Compute final dose = ON

4^th^ round

- Optimization tolerance: 1.000E-9
- Max number of iterations:300
- Iteration before conversion:100
- Compute intermediate dose = ON
- Compute final dose = ON

Scaled dose

- ROI = PTV-air
- Dose at volume = 95%
- Dose = 4600

**Objective/Constraints for initial plans**

| **ROI** | **Description** | **Value** | **Weight** |
| --- | --- | --- | --- |
| Dose line 100%RX | Min dose | 4600cGy | 10 |
| Dose line 100%RX | Max dose | 4650cGy | 10 |
| Dose line 100%RX | Uniform dose | 4600cGy | 10 |
| Dose line 100%RX | Max EUD | 4680cGy, ParameterA = 150 | 10 |
| Ring("Dose line 100%RX" +5mm - "Dose line 100%RX"+1mm) | Max dose | 4600cGy | 10 |
| Body-5mm ^ (Dose line 90%RX - Dose line 95%RX) | Max dose | 4370cGy | 0.1 |
| Body-5mm ^ (Dose line 80%RX - Dose line 90%RX) | Max dose | 4140cGy | 0.1 |
| Body-5mm ^ (Dose line 70%RX - Dose line 80%RX) | Max dose | 3680cGy | 0.1 |
| Body-5mm ^ (Dose line 60%RX - Dose line 70%RX) | Max dose | 3220cGy | 0.1 |
| Body-5mm ^ (Dose line 50%RX - Dose line 60%RX) | Max dose | 2760cGy | 0.1 |
| Body-5mm ^ (Dose line 400%RX - Dose line 50%RX) | Max dose | 2300cGy | 0.1 |
| Body-5mm ^ (Dose line 30%RX - Dose line 40%RX) | Max dose | 1840cGy | 0.1 |
| Body-5mm ^ (Dose line 20%RX - Dose line 30%RX) | Max dose | 1380cGy | 0.1 |
| Body-5mm ^ (Dose line 10%RX - Dose line 20%RX) | Max dose | 920cGy | 0.1 |
| Body-5mm ^ (Dose line 5%RX - Dose line 10%RX) | Max dose | 460cGy | 0.1 |
| OAR - Dose line 30%RX | Max dose | 1380cGy | 0.01 |
| OAR - Dose line 20%RX | Max dose | 920cGy | 0.01 |

**B. Optimization settings for boost plans**

1^st^ round

- Optimization tolerance: 1.000E-9
- Max number of iterations:400
- Iteration before conversion:100
- Compute intermediate dose = ON
- Compute final dose = ON

2^nd^ round

- Optimization tolerance: 1.000E-9
- Max number of iterations:300
- Iteration before conversion:100
- Compute intermediate dose = ON
- Compute final dose = ON

3^rd^ round

- Optimization tolerance: 1.000E-9
- Max number of iterations:200
- Iteration before conversion:100
- Compute intermediate dose = ON
- Compute final dose = ON

Scaled dose

- ROI = Dose line 100%RX
- Dose at volume = 98%
- Dose = 2400

4^th^ round

- Optimization tolerance: 1.000E-9
- Max number of iterations:400
- Iteration before conversion:100
- Compute intermediate dose = ON
- Compute final dose = ON

5^th^ round

- Optimization tolerance: 1.000E-9
- Max number of iterations:300
- Iteration before conversion:100
- Compute intermediate dose = ON
- Compute final dose = ON

6^th^ round

- Optimization tolerance: 1.000E-9
- Max number of iterations:200
- Iteration before conversion:100
- Compute intermediate dose = ON
- Compute final dose = ON

Scaled dose

- ROI = PTV-air
- Dose at volume = 95%
- Dose = 2400

**Objective/Constraints for boost plans**

| **ROI** | **Description** | **Value** | **Weight** |
| --- | --- | --- | --- |
| Dose line 100%RX | Min dose | 2400cGy | 10 |
| Dose line 100%RX | Max dose | 2450cGy | 10 |
| Dose line 100%RX | Uniform dose | 2400cGy | 10 |
| Dose line 100%RX | Max EUD | 2470cGy,  ParameterA = 150 | 10 |
| Ring("Dose line 100%RX" +5mm - "Dose line 100%RX"+1mm) | Max dose | 2400cGy | 10 |
| Body-5mm ^ (Dose line 90%RX - Dose line 95%RX) | Max dose | 2280cGy | 5 |
| Body-5mm ^ (Dose line 80%RX - Dose line 90%RX) | Max dose | 2160cGy | 5 |
| Body-5mm ^ (Dose line 70%RX - Dose line 80%RX) | Max dose | 1920cGy | 5 |
| Body-5mm ^ (Dose line 60%RX - Dose line 70%RX) | Max dose | 1680cGy | 5 |
| Body-5mm ^ (Dose line 50%RX - Dose line 60%RX) | Max dose | 1440cGy | 5 |
| Body-5mm ^ (Dose line 400%RX - Dose line 50%RX) | Max dose | 1200cGy | 5 |
| Body-5mm ^ (Dose line 30%RX - Dose line 40%RX) | Max dose | 960cGy | 5 |
| Body-5mm ^ (Dose line 20%RX - Dose line 30%RX) | Max dose | 720cGy | 5 |
| Body-5mm ^ (Dose line 10%RX - Dose line 20%RX) | Max dose | 480cGy | 5 |
| Body-5mm ^ (Dose line 5%RX - Dose line 10%RX) | Max dose | 240cGy | 5 |
| OAR - Dose line 30%RX | Max dose | 720cGy | 0.01 |
| OAR - Dose line 20%RX | Max dose | 480cGy | 0.01 |
